# Protocol for a systematic review of wearable devices for antenatal fetal monitoring

**DOI:** 10.12688/wellcomeopenres.24335.1

**Published:** 2025-07-23

**Authors:** Niccole Ranaei-Zamani, Olayinka Kowobari, Dimitrios Siassakos, Sara Hillman, Anna L David, Subhabrata Mitra

**Affiliations:** 1 Neonatology, Elizabeth Garrett Anderson Institute for Women’s Health, University College London, London, England, WC1E 6DE, UK; 2 Maternal and Fetal Medicine, Elizabeth Garrett Anderson Institute for Women’s Health, University College London, London, England, UK

**Keywords:** Wearable devices, fetal monitoring, antenatal care, pregnancy outcomes, remote health monitoring, maternal health technology, systematic review

## Abstract

**Introduction:**

Fetal monitoring is a crucial component of antenatal care, facilitating early detection of fetal compromise and improving pregnancy outcomes. Traditional monitoring methods such as cardiotocography (CTG) and ultrasound are effective but primarily limited to clinical settings, requiring specialized expertise and resources. The rise of wearable medical devices and artificial intelligence (AI) applications presents an opportunity to enhance fetal monitoring by enabling continuous, real-time data collection outside clinical environments. These technologies have the potential to improve fetal health and obstetric outcomes, particularly in resource-limited settings. This systematic review aims to evaluate the use of wearable devices for antenatal fetal monitoring and their impact on fetal and obstetric outcomes.

**Methods and Analysis:**

This systematic review will adhere to the Preferred Reporting Items for Systematic Reviews and Meta-Analyses (PRISMA) guidelines and the Synthesis Without Meta-analysis (SWiM) framework. A comprehensive search of PubMed, Embase, Cochrane Library, and Web of Science will be conducted to identify primary research studies investigating wearable devices designed for fetal monitoring during pregnancy. Studies will be included if they assess the effectiveness, accuracy, and clinical impact of wearable fetal monitoring devices. Primary outcomes will include markers of fetal well-being as well as neonatal and obstetric outcomes. Secondary outcomes will focus on patient experience and acceptability. Data extraction and quality assessment will be conducted independently by two reviewers using the National Institutes of Health (NIH) Quality Assessment Tool and the Newcastle-Ottawa Scale. A narrative synthesis will be performed to summarise the findings.

**Ethics and Dissemination:**

Ethical approval is not required since the study involves analysing published literature. The findings will be shared through peer-reviewed publications and conference presentations. This review will enhance the evidence base regarding the clinical utility of wearable fetal monitoring technologies and inform future research and device development. PROSPERO Registration: CRD4202348755 (current version 4.1)

## Plan language summary

During pregnancy, keeping track of a baby’s health is important for spotting problems early and improving outcomes. At the moment, most monitoring is done in hospitals using machines that required trained staff and regular clinic visits. But new wearable devices – like smart patches or belts – could change that. These devices are small, portable, and can monitor the baby’s wellbeing at home, making pregnancy care more accessible and less stressful for patients.

In this study, we plan to carefully review all the scientific research that has tested these wearable fetal monitoring devices during pregnancy. We will look at how well these devices work, what health outcomes they affect, and how pregnant people feel about using them. We will include studies from around the world, across all types of healthcare settings, to get a full picture.

By bringing together the evidence, we aim to understand whether these technologies could help improve care during pregnancy, especially in places where access to clinics is limited. This review will also help guide future research and design of pregnancy monitoring tools that are safe, effective, and acceptable to patients.

## Strengths and limitations of this study

- This is the first systematic review to our knowledge explicitly investigating the study and development of wearable devices in the antenatal period to monitor fetal wellbeing.
- This study will also attempt to collate qualitative feedback from pregnant patients which will be important due to specific design requirements needed for the development of wearable devices for use in pregnancy, which will provide guidance for future design of future clinical and commercial devices
- This review will be conducted by undertaking a comprehensive review of several databases to ensure data is collected from a range of sources.
- This study may be limited by the lack of randomised controlled trials due to the potential unacceptable risk and ethical considerations of exposing pregnant patients to novel forms of fetal monitoring without large quantities of robust observational data to suggest benefit or equivalence over traditional fetal monitoring.
- Due to the likely heterogeneity of studies and technologies, it is not possible at this stage to conduct a meta-analysis of the data, but will complete a descriptive narrative review of the available data.

## Introduction

Fetal monitoring is a fundamental element of antenatal care, enabling the early detection of fetal compromise and improving pregnancy outcomes. In the USA, stillbirth affects approximately 1 in 175 pregnancies (stillbirth rate (SBR) of 5.7/1000 births) ^1^. Although stillbirth rates in the UK are lower (approximately 1 in every 250 births, SBR 3.8/1000 births) ^2^, this rate has remained relatively static despite increased funding for maternity services and the NHS’s ‘Saving Babies’ Lives Care Bundle,’ which aims to halve the number of stillbirths by 2030 ^3^. This rate remains higher than in many comparable European countries, underscoring the need for more effective interventions and resources. Globally, stillbirth disproportionately affects low- and middle-income countries (LMICs), with India reporting the highest number of stillbirths—340,600 in 2019, equating to a stillbirth rate (SBR) of 13.9 per 1,000 births. In 2023, ^4^50% of the total of all stillbirths worldwide occurred in sub-Saharan Africa (SBR 22.2 across the entire region) ^4^.

These disparities underscore the critical need for scalable, accurate, and affordable tools to expand access to high-quality antenatal care and address inequities in fetal outcomes.

Traditional antenatal fetal monitoring methods, such as cardiotocography (CTG) and ultrasound, are primarily limited to clinical settings, requiring significant resources and specialised expertise. The COVID-19 pandemic accelerated advancements in telemedicine, wearable medical devices, and artificial intelligence (AI) applications, creating unprecedented opportunities to revolutionise antenatal fetal monitoring. These innovations enable real-time, continuous monitoring outside clinical environments, offering the potential to improve fetal health outcomes, particularly in underserved regions.

This review focuses on the use of wearable medical devices for antenatal fetal monitoring, distinct from maternal health monitoring or intrapartum care. By leveraging cutting-edge artificial intelligence (AI) and machine learning (ML) technologies, these devices promise to enhance the accuracy, accessibility, and affordability of fetal monitoring. This study evaluates their impact on fetal and obstetric outcomes, aiming to highlight the transformative potential of these innovations in reducing global disparities in antenatal care.

## Objectives

We aim to systematically evaluate the findings of studies investigating the use and development of wearable devices for fetal monitoring in pregnancy during the antenatal period to assess the impact of these on fetal and obstetric outcomes in pregnancy. To achieve this, the following key ideas will be addressed:

1. The different technologies being explored
2. The impact of these technologies on fetal outcomes
3. The impact of these technologies on obstetric outcomes
4. Qualitative data regarding acceptability for patients of wearable medical devices in pregnancy
5. To assess newer technologies with the capability for automation with machine learning and smartphone integration

## Methods

This protocol will follow the applicable sections of the ‘PRISMA (Preferred Reporting Items for Systematic Reviews and Meta-Analyses) protocol guidelines’ ^5, 6^ and the ‘Synthesis Without Meta-analysis (SWiM) in Systematic Reviews reporting guidelines’ ^7^. This systematic review is registered with PROSPERO (the International Prospective Register of Systematic Reviews under registration number CRD42023487557).

### Patient population

Pregnant patients across all gestations will be included in this review. There will be no restrictions based on geographical location. We will include studies from all resource settings. There will also be no restrictions on risk status or underlying maternal medical conditions.

### Interventions

Interventions must be wearable medical devices specifically for use in the antenatal period for fetal monitoring. These can be wearable devices which have been trialled within the hospital setting or the community (including use at home), which allow for ambulatory monitoring. We will also include any interventions utilising machine learning algorithms for data analysis derived from wearable devices **.**

### Primary outcomes

All pregnancy outcome measures directly reflecting fetal health, neonatal outcomes and obstetric parameters will be extracted from the studies.

1. Fetal wellbeing
  a. Fetal heart rate variability
  b. Fetal movement patterns and frequency
  c. Fetal biophysical profiles
  d. Parameters indicative of fetal distress or compromised well-being
2. Neonatal outcomes
  a. Birth weight
  b. Gestational age at birth
  c. Apgar scores at delivery
  d. Incidence of low birth weight or macrosomia
  e. Incidence of intrauterine growth restriction (IUGR)
  f. Fetal distress during labour or delivery
  g. Adverse fetal outcomes: neonatal encephalopathy, hypoxic brain injury, need for neonatal resuscitation, need for respiratory support or neonatal intensive care unit (NICU) admissions
3. Obstetric outcomes
  a. Incidence of preterm delivery
  b. Incidence of pre-eclampsia
  c. Incidence of gestational hypertension
  d. Maternal complications during labour or delivery (e.g. PPH)
  e. Mode of delivery

### Secondary outcomes

1. Patient experience
  a. Patient feedback on acceptability and experience using the device: Quantitative or qualitative data

### Eligibility criteria

The following criteria will be used to evaluate the suitability of studies for inclusion in the literature review by following the PICO framework:

***Inclusion criteria***

- Primary research studies and Clinical investigations (clinical studies, prospective clinical studies, randomised clinical trials, retrospective review of clinical data)
- Availability of abstract and full text in English
- Wearable devices for fetal monitoring in the antenatal period
- Sources published between 2000 up until the date of the search
- Studies involving pregnant patients above the age of 18, in any country

***Exclusion criteria***

- Studies involving prototypes not trialled in human patients (i.e. phantom or computer modelling studies)
- Systematic Reviews and Meta-Analyses
- Devices intended only for intrapartum use

## Search strategy

### Electronic databases

We performed a systematic search of the following online databases: Pubmed, Cochrane Library, Embase, and Web of Science. The search will follow the PRISMA search strategy. Only English language publications published between 1 ^st^ January 2000 and 31 ^st^ January 2025 will be included, as it felt that we were unlikely to find relevant advanced technologies prior to 2000. The references of any systematic reviews found will also be screened; however, systematic reviews themselves will not be included in this review to ensure the synthesis of original clinical data.

This review will focus exclusively on primary clinical studies that evaluated the use of wearable devices for fetal monitoring in antenatal ambulatory settings. However, as this review concentrates on medical devices, any studies investigating the technical development of devices (such as lab testing) will be included as long as there is evidence of data of use on patients.

The search terms will be identified using a combination of keywords and Medical Subject Heading (MeSH) terms contained in titles.

Keywords include phrases such as:

- **Wearable devices:** “Wearable technology”, “Wearable sensors”, “Remote monitoring” and “Wearable health devices”
- **Pregnancy:** “Pregnant women”, “Antenatal care” and “Gestational health”
- **Fetal and obstetric outcomes:** “Fetal health”, “Neonatal outcomes”, “Pregnancy outcomes”, “Birth outcomes”, “Obstetric complications” and “Stillbirth”

Keywords and MesH terms will then be integrated using Boolean operators (AND, OR) to create search strings. For example (“Wearable devices” OR “Wearable sensors”) AND (“Pregnancy” OR “Antenatal care”) AND (“Fetal outcomes OR “Birth outcomes”).

### Study selection

Studies identified through searches of electronic databases and manual searches will undergo a two-stage selection process: firstly by title and abstract screening and then by full-text screening. Study selection will be independently performed by two reviewers (NRZ and OK) using the selection criteria discussed above. Any discrepancies between reviewers will be discussed, and if consensus cannot be reached, a third reviewer (SM) will be consulted. 

### Data extraction

Data extraction will be performed by two independent reviewers (NRZ and OK). The first reviewer will extract the relevant data using a standardised format, while the second reviewer will verify the extracted data for accuracy. Any discrepancies will be resolved through discussion, and if a consensus, a third reviewer (SM) will be consulted. If key data are missing or required clarification, the study authors will be contacted. 

Key data to be extracted will include:

- Authors
- Article title
- Journal
- Year of publication
- Country
- Study design
- Population (including any gestational ranges or risk status)
- Site of monitoring (e.g. home, hospital or laboratory)
- Number of pregnant patients included
- Key outcomes relating to performance of device (e.g. signal acquisition, comparability to gold standard)
- Key outcomes relating to clinical outcomes (fetal and obstetric outcomes)
- Key outcomes related to patient experience obtained through qualitative measures

### Quality assessment and risk of bias tools

Each study will be quality assessed using either the National Institute for Health (NIH) Quality Assessment Tool for Observational Cohort and Cross-Sectional Studies ^8^ or the Newcastle-Ottawa Scale ^9^ depending on the study type and design. Two reviewers (NRZ and OK) will independently assess the quality of each included study. Any discrepancies will be discussed and resolved via consensus or through the involvement of a third reviewer (SM). The quality assessment for each study will be presented in a table for inclusion in the review.

### Data synthesis

Once the study selection process is complete, the process will be presented in a PRISMA flowchart ^10^. The final studies included will be categorised by the type of fetal monitoring device if possible. We will exclude any studies found to have a high risk of bias. A meta-analysis will not be conducted due to anticipated heterogeneity in the device types and outcomes. A narrative synthesis of the results will be conducted by using the *Popay et al. ^11^* framework. The structure of the narrative synthesis will predominantly revolve around the type of device used in the study, the performance of the device, comparisons to current standards of care and evidence of acceptability by pregnant patients.

### Patient and public involvement/ethics

No patients were involved in this systematic review and we will only include data which has already been published, therefore ethical approval was not obtained.

## Dissemination

The findings of the systematic review will be published in an open-access peer-reviewed journal. This systematic review will be used to develop a qualitative study to understand patient perspectives towards a new optical fetal monitoring device which is being developed and to guide the design of future clinical trials for this device.

## Discussion

Several previous systematic reviews have investigated wearable devices for monitoring maternal health. To our knowledge, this is the first systematic review which focuses solely on wearable fetal monitoring devices exclusively in the antenatal period.

We are particularly interested in investigating studies which have considered the cost and scalability of these devices since LMICs share a disproportionate burden of poor fetal and obstetric outcomes. Almost 80% of all global stillbirths occurred in South Asia and sub-Saharan Africa ^4^. Currently, many LMICs lack access to basic obstetric care and therefore integration of these new technologies must also consider the existing healthcare infrastructure and how this can support these new monitoring tools.

An anticipated limitation is there is likely to be a low number of RCTs collated as part of this review, as many of these technologies are still emerging and undergoing technological advancements, and therefore may still be in the earlier stages of the clinical trial process. Lastly, pregnancy can be an anxiety-inducing life event for women of any risk status, therefore the development and implementation of novel technologies must be conducted with involvement of pregnant women themselves. Therefore, being able to also evaluate the patient perspectives of using these devices will help to inform future device development in this field.

## Data Availability

Not applicable as no datasets produced for this study. PRISMA-P checklist available on Zenodo, ‘PRISMA-P checklist for ‘Protocol for a systematic review of wearable devices for antenatal monitoring’, DOI: https://doi.org/10.5281/zenodo.15553579, under a CC0 licence ^6^.

## References

[1] CDC: U.S. Centres for Disease Prevention and Control.2024. Reference Source

[2] Office for National Statistics: Births in England and Wales: 2022.2023. Reference Source

[3] O’Connor D : Saving babies’ lives: a care bundle for reducing stillbirth.2016. Reference Source

[4] United Nationals Inter-agency Group for Child Mortality Estimation, 2024: Standing up for stillbirth: current estimates and key interventions.2025. Reference Source

[5] Moher D Shamseer L Clarke M : Preferred Reporting Items for Systematic review and Meta-Analysis Protocols (PRISMA-P) 2015 statement. *Revista Espanola de Nutricion Humana y Dietetica.* 2016;20(2):148–160.

[6] Ranaei-Zamani N : PRISMA-P checklist for ‘Protocol for a Systematic Review of Wearable Devices for Antenatal Fetal Monitoring’. *Zenodo.* 2025. 10.5281/zenodo.15553580 PMC12635520 41281953

[7] Campbell M McKenzie JE Sowden A : Synthesis Without Meta-analysis (SWiM) in systematic reviews: reporting guideline. *BMJ.* 2020;368: l6890. 10.1136/bmj.l6890 31948937 PMC7190266

[8] National Heart, Lung and Blood Institute: Study Quality Assessment Tools.2021. Reference Source

[9] Wells G Shea B O'Connell D : The Newcastle-Ottawa Scale (NOS) for assessing the quality of nonrandomised studies in meta-analyses. Reference Source

[10] PRISMA 2020: PRISMA flow diagram. Reference Source

[11] Popay J Arai L Rodgers M : Guidance on the conduct of narrative synthesis in systematic reviews: a product from the ESRC methods programme.2006. 10.13140/2.1.1018.4643

