# Supplementary material for: Protocol for a systematic review of wearable devices for antenatal fetal monitoring
Source: Wellcome Open Res. 2025 Oct 31;10:356. Originally published 2025 Jul 23. [Version 2] doi: 10.12688/wellcomeopenres.24335.2 (PMC12635520; doi:10.12688/wellcomeopenres.24335.2)
Supplement: Peer review history

# Reviewer report: Reviewer response for version 2

**DOI:** 10.21956/wellcomeopenres.27829.r138090

**Published:** 2025-12-26

**Authors:** Benjamin Smarr

**Affiliations:** 1 University of California, ,San Diego, California, USA

The authors have done a nice job of revising the comments. Glad they were helpful. As the other reviewer observed, there's always some small proofing to catch, but I am not particularly concerned with that issue here.

Is the study design appropriate for the research question?

Partly

Is the rationale for, and objectives of, the study clearly described?

Partly

Are sufficient details of the methods provided to allow replication by others?

No

Are the datasets clearly presented in a useable and accessible format?

Not applicable

Reviewer Expertise:

on and near body sensors, multimodal data, women's health and reproductive neuroendocrinology, healthAI

I confirm that I have read this submission and believe that I have an appropriate level of expertise to confirm that it is of an acceptable scientific standard.

# Reviewer report: Reviewer response for version 2

**DOI:** 10.21956/wellcomeopenres.27829.r138088

**Published:** 2025-11-22

**Authors:** Corinne Caillaud

**Affiliations:** 1 Faculty of Medicine and Health, The University of Sydney, Sydney, Australia

I appreciate the authors’ responses to my comments and the revisions made to the manuscript as suggested.

 My only remaining comment on version 2 concerns the consistency of verb tense. Please review the manuscript and ensure that tense is consistent throughout.

Is the study design appropriate for the research question?

Partly

Is the rationale for, and objectives of, the study clearly described?

Partly

Are sufficient details of the methods provided to allow replication by others?

Yes

Are the datasets clearly presented in a useable and accessible format?

Not applicable

Reviewer Expertise:

Digital health and wearables for health promotion and disease prevention - Young people -

I confirm that I have read this submission and believe that I have an appropriate level of expertise to confirm that it is of an acceptable scientific standard.

# Reviewer report: Reviewer response for version 2

**DOI:** 10.21956/wellcomeopenres.27829.r138089

**Published:** 2025-11-20

**Authors:** Ha Uk Chung

**Affiliations:** 1 Korea University, Seongbuk-gu, Seoul, South Korea

The author made a strong amendment that meets the expectation for submission. Thanks to authors for the works and I am happy to approve the submission.

Is the study design appropriate for the research question?

Yes

Is the rationale for, and objectives of, the study clearly described?

Yes

Are sufficient details of the methods provided to allow replication by others?

Yes

Are the datasets clearly presented in a useable and accessible format?

Yes

Reviewer Expertise:

Medical device research, development, deployment, regulatory process, and industrialization.

I confirm that I have read this submission and believe that I have an appropriate level of expertise to confirm that it is of an acceptable scientific standard.

# Reviewer report: Reviewer response for version 1

**DOI:** 10.21956/wellcomeopenres.26836.r134630

**Published:** 2025-10-25

**Authors:** Ha Uk Chung

**Affiliations:** 1 Korea University, Seongbuk-gu, Seoul, South Korea

This protocol presents a well-organized plan for a systematic review of advanced wearable technologies in fetal monitoring during antenatal period. The topic has a clear clinical, technical, and societal impact when done properly. While authors provide a clear plan, there are some minor comments. 

   1) Definition and Scope of Wearable Devices are unclear. Wearable devices may exist as a medical device with an appropriate regulatory approval or as a consumer devices, or even as a research version. Authors need to define the scope more clearly. 

   2) The analysis plan is a bit obscure, especially with the long list of fetal, neonatal, and obstetric outcomes. Authors may consider give a priority in the list, given the database may not have a full data point. 

   3) LMIC - authors show interest in exploring LMIC context, however there are no clear description or plan. 

 After the above comments are fulfilled, the protocol should be indexable.

Is the study design appropriate for the research question?

Yes

Is the rationale for, and objectives of, the study clearly described?

Yes

Are sufficient details of the methods provided to allow replication by others?

Yes

Are the datasets clearly presented in a useable and accessible format?

Yes

Reviewer Expertise:

Medical device research, development, deployment, regulatory process, and industrialization.

I confirm that I have read this submission and believe that I have an appropriate level of expertise to confirm that it is of an acceptable scientific standard, however I have significant reservations, as outlined above.

# Response

**Published:** 2025-10-27

**Authors:** Niccole Ranaei-Zamani

**Affiliations:** Neonatology, University College London, London, England, UK

We thank the Reviewer for the positive and constructive comments, which have helped to further refine the clarity and focus of the protocol.

 We have clarified the definition and scope of wearable devices to specify that only medical-grade or research-validated on-body sensors designed for clinical or research use during the antenatal period are included. This ensures that the scope is clearly delineated between clinical, research, and consumer-grade technologies. We acknowledge the suggestion to prioritise among the large number of potential fetal, neonatal, and obstetric outcomes. The revised Methods section now specifies that primary emphasis will be placed on fetal well-being parameters such as fetal heart rate, movement, and biophysical indicators, with neonatal and obstetric outcomes summarised where available. This provides greater focus and transparency in the analysis plan. We have also incorporated the reviewer’s feedback regarding the low- and middle-income country (LMIC) context. The Methods section now specifies that information on study setting and resource context (for example, high-income versus low- or middle-income countries) will be extracted where reported. This will allow interpretation of cost, scalability, and implementation potential across different healthcare environments. We appreciate the reviewer’s recognition of the clinical and societal relevance of this topic. These revisions further strengthen the precision, coherence, and applicability of the protocol.

# Reviewer report: Reviewer response for version 1

**DOI:** 10.21956/wellcomeopenres.26836.r134628

**Published:** 2025-10-20

**Authors:** Benjamin Smarr

**Affiliations:** 1 University of California, ,San Diego, California, USA

This short prospectus outlines what would become a review of technology aimed at fetal monitoring. The authors describe a focus on scalability and access for underserved populations, which I applaud.

 - I would encourage the authors to expand the introduction, as fetal loss is far from the only concern to support the need for monitoring technologies.

 - Devices do not use AI. Devices generate data on which AI can be run, so it is the devices and appropriate AI services that hold promise, not just the devices themselves. This matters because access to data and or transparency/explainability/verifiability of algorithms on diverse populations are critical to measuring the actual impact of these services on different populations.

 - I am not sure what level of analytic specificity is expected, but I would encourage the authors to explicitly state that they will break down analyses by covariates such as region, age, gestational state, etc., as answers likely vary across contexts. One lump average is less likely to be useful.

 - I encourage the authors to expand their search criteria to include things like “on body sensor,”, “dermal [or] skin sensor” etc., as not all on body technologies call themselves “wearable”.

Is the study design appropriate for the research question?

Partly

Is the rationale for, and objectives of, the study clearly described?

Partly

Are sufficient details of the methods provided to allow replication by others?

No

Are the datasets clearly presented in a useable and accessible format?

Not applicable

Reviewer Expertise:

on and near body sensors, multimodal data, women's health and reproductive neuroendocrinology, healthAI

I confirm that I have read this submission and believe that I have an appropriate level of expertise to confirm that it is of an acceptable scientific standard, however I have significant reservations, as outlined above.

# Response

**Published:** 2025-10-27

**Authors:** Niccole Ranaei-Zamani

**Affiliations:** Neonatology, University College London, London, England, UK

We thank the Reviewer for the constructive and insightful feedback, which has strengthened the technical precision and clarity of our protocol.

 We have clarified the scope of the review and refined the terminology used to describe wearable technologies. The text now distinguishes clearly between the devices that generate physiological data and any subsequent analytical or artificial-intelligence-based interpretation of those data. The review therefore evaluates the performance and feasibility of the wearable devices themselves rather than algorithms as independent interventions. The Introduction has been expanded to acknowledge that fetal loss is only one of several clinical motivations for antenatal monitoring. The revised text now highlights broader objectives, including detection and management of complications such as fetal growth restriction, pre-eclampsia, preterm labour, and fetal distress. We appreciate the reviewer’s suggestion regarding subgroup analyses by region, age, or gestational state. After reviewing the available evidence, we concluded that such analyses would not be feasible due to heterogeneity in study design and outcome reporting. This has been clarified in the Methods section. While the overall search structure remains as initially designed, it already captures studies using a broad range of terminology relevant to wearable or on-body sensing technologies. We have also emphasised considerations of scalability and accessibility, particularly for low- and middle-income settings, as these issues are central to the potential impact of wearable fetal monitoring technologies. We thank the reviewer for these valuable suggestions, which have helped refine the conceptual and analytical framing of the protocol.

# Reviewer report: Reviewer response for version 1

**DOI:** 10.21956/wellcomeopenres.26836.r128378

**Published:** 2025-08-29

**Authors:** Corinne Caillaud

**Affiliations:** 1 Faculty of Medicine and Health, The University of Sydney, Sydney, Australia

This article outlines the protocol for a systematic review aimed at evaluating the use of wearable devices for antenatal fetal monitoring and their impact on fetal outcomes. The potential of wearable technology to enhance fetal well-being is promising; however, it is essential to assess its indications and effectiveness in improving pregnancy outcomes. This review has the potential to identify key factors contributing to effectiveness and to inform clinical practice.

 Nonetheless, several aspects require clarification. In particular, the article would benefit from a more clearly defined research question and stronger alignment between the research question, main objectives, and methodology. Additionally, the secondary outcome—acceptability—may not be consistently addressed across the included studies.

 Abstract:

 In the introduction, it will be beneficial to define wearable devices in the context of your study and to better characterise the type of wearable you are interested in. Are these wearables used as consumer technology or clinical monitoring based on specific identified risk(s) for the foetus?

 Plain language summary: monitoring is done in hospital using machines": please consider to briefly indicate what these machines measure.

 Strength and limitations:

- First sentence. Are you investigating the development of the wearable or their effectiveness in assessing target fetal health outcomes? From this abstract it sounds that the review does not assess the development of wearables. Please clarify.
- Second sentence: in line with my comment above, please clarify how the qualitative feedback will be collated.

 

 I would recommend making the strengths and limitations statements more concise.

**Introduction**

 The introduction is concise and well written; however it lacks details on the type of wearable monitoring devices this work will review. The introduction must provide additional information about the wearables and what are the clinical indications (this will guide the assessment of effectiveness). It would also be helpful to identify the main variable outcomes that can be assessed by these wearables, such as fetal heart rate or movement for example.

 Similarly, it is expected that the objectives identify more precisely the fetal outcomes of interest. Providing more information about the wearable devices will help identify these outcomes because most existing technologies assess fetal heart rate, movement and patients heart rate, body temperature, oxygen transport and contractions. Are all of these of interest?

 Qualitative feedback is again mentioned in the objectives but not introduced. Indicating the type of outcomes investigated (acceptability for example) is important because it will strengthen the rational for the review.

**Objectives.**

 The objectives are somewhat misaligned with the introduction, making it  difficult to follow the main objective of the review. Clarifying your research question in the introduction will help strengthening the alignment of objectives.

 For example, you say in 1) that the review will address the different technologies being explored but in method you indicate that you will only include medical devices. A medical device is not just exploratory but validated. You also said that the aim was to assess the effectiveness not just explore the different technologies. The aim set in the introduction is good and the authors can just align with it.

 If the evidence of any utility of these wearables for fetal and obstetric outcomes are not known, this review is very important. This one objective may be enough.

**Methodology**

 The protocol includes an extensive list of primary outcome measures, and it would be beneficial to clarify how these outcomes align with the research question or the clinical indications for monitoring. In the methods section, secondary outcomes are defined in terms of patient acceptability based on user experience. However, it is unclear whether this information will be extracted from the same pool of studies as those addressing the primary outcomes. Given that the methodologies for assessing primary and secondary outcomes may differ, they are likely to be reported in distinct sets of articles.

 The selected keywords appropriately reflect the scope of the primary outcomes. Nonetheless, it should be noted that data related to the secondary outcome may be incomplete or inconsistently reported across the included studies. Overall, the remainder of the methodology appears sound.

**Discussion**

 The authors acknowledge the existence of several prior reviews in the same area of interest. To strengthen the rationale for this review, it would be beneficial to reference these earlier works and clearly articulate how the current review differs—particularly in terms of scope, methodology, or the novelty of its contributions.

 The discussion highlights an interest in studies addressing cost and scalability, which are indeed critical considerations. However, these aspects are not introduced earlier in the manuscript. If cost and scalability are intended to be focal points, they should be explicitly mentioned in the introduction and reflected in the selection criteria.

 In conclusion, this review addresses an important topic, but revisions are needed to ensure consistency and alignment of the research questions and objectives throughout the introduction, methodology, and discussion sections.

Is the study design appropriate for the research question?

Partly

Is the rationale for, and objectives of, the study clearly described?

Partly

Are sufficient details of the methods provided to allow replication by others?

Yes

Are the datasets clearly presented in a useable and accessible format?

Not applicable

Reviewer Expertise:

Digital health and wearables for health promotion and disease prevention - Young people -

I confirm that I have read this submission and believe that I have an appropriate level of expertise to confirm that it is of an acceptable scientific standard, however I have significant reservations, as outlined above.

# Response

**Published:** 2025-10-27

**Authors:** Niccole Ranaei-Zamani

**Affiliations:** Neonatology, University College London, London, England, UK

We thank the Reviewer for the thoughtful and constructive feedback, which has helped strengthen the clarity and alignment of our protocol.

 We have revised the research question, objectives, and methodology to ensure complete consistency throughout the manuscript. The research question now clearly focuses on the effectiveness and acceptability of wearable medical devices for antenatal fetal monitoring, and the objectives have been refined to mirror this focus. The Methods section has been updated so that all planned analyses directly address these aims. The definition and scope of “wearable devices” have been clarified. The review now specifies inclusion of medical-grade or research-validated on-body sensors designed for antenatal fetal monitoring, while explicitly excluding consumer wellness or intrapartum devices. This clarification has been added to both the Introduction and Methods. The description of the secondary outcome (acceptability) has been expanded. The Methods section now states that qualitative and quantitative data on user experience and acceptability will be extracted where reported and synthesised descriptively, acknowledging that such data are often presented separately from clinical outcomes. The list of outcome measures has been refined and prioritised. Primary emphasis will be placed on fetal well-being indicators such as heart-rate variability and movement, while neonatal and obstetric outcomes will be summarised where available to maintain coherence with the central research question. The Discussion has been expanded to explain how this review differs from prior work, emphasising that it is the first to focus exclusively on antenatal wearable fetal monitoring technologies. References to cost and scalability have been integrated throughout to strengthen the global and implementation context. We thank the reviewer again for the insightful comments, which have led to significant improvements in clarity, focus, and overall structure of the protocol.
